# Is Intergenerational Social Mobility Related to the Type and Amount of Physical Activity in Mid-Adulthood? Results from the 1946 British Birth Cohort Study

**DOI:** 10.1016/j.annepidem.2012.03.002

**Published:** 2012-07

**Authors:** Richard J. Silverwood, Mary Pierce, Dorothea Nitsch, Gita D. Mishra, Diana Kuh

**Affiliations:** a Department of Non-Communicable Disease Epidemiology, Faculty of Epidemiology and Population Health, London School of Hygiene and Tropical Medicine, London, UK; b MRC Unit for Lifelong Health and Ageing, University College London, London, UK; c School of Population Health, University of Queensland, Brisbane, Australia

**Keywords:** Cohort Study, Education, Exercise, Leisure Activities, Occupation, Physical Activity, Social Mobility, Socioeconomic Position, CVD, cardiovascular disease, LCA, latent class analysis, LTPA, leisure-time physical activity, SEP, socioeconomic position

## Abstract

**Purpose:**

Greater levels of leisure-time or moderate-vigorous physical activity have consistently been found in those with greater socioeconomic position (SEP). Less is known about the effects of intergenerational social mobility.

**Methods:**

We examined the influence of SEP and social mobility on mid-adulthood physical activity in the Medical Research Council National Survey of Health and Development. Two sub-domains of SEP were used: occupational class and educational attainment. Latent classes for walking, cycling, and leisure-time physical activity (LTPA) were used, plus sedentary behavior at age 36. Associations between types of physical activity and SEP were examined with the use of logistic or multinomial logistic regression.

**Results:**

Being a manual worker oneself or having a father who was a manual worker was, relative to nonmanual work, associated with lower levels of sedentary behavior and greater walking activity, but also with lower LTPA. Compared with those who remained in a manual occupational class, upward occupational mobility was associated with more sedentary behavior, less walking, and increased LTPA. Associations with downward mobility were in the opposite directions. Similar results were obtained for educational attainment.

**Conclusions:**

This study found clear evidence of social differences in physical activity. Persistently high SEP and upward social mobility were associated with greater levels of LTPA but also increased sedentary behavior and less walking.

## Introduction

Epidemiological evidence has confirmed the benefits of regular physical activity on health and well-being (1). Promoting a physically active lifestyle is now considered a major element of public health policies, and increases in leisure-time physical activity (LTPA) have been reported in some countries (2, 3).

Recently, time spent in sedentary behavior, as defined by prolonged sitting or reclining characterized by low energy expenditure, has been shown to be associated with obesity (4–6), metabolic syndrome (7, 8), type 2 diabetes mellitus (5, 9), markers of cardiovascular disease (CVD) risk (4), and all-cause and CVD mortality (10), independently of levels of physical activity.

Greater levels of leisure-time or moderate-vigorous activity have consistently been found in those with greater socioeconomic position (SEP) (11). However, most studies to date have focused on LTPA only (12). In addition, many studies of physical activity and SEP use only a single subdomain of SEP (11), reducing the robustness of their conclusions.

Little is known about the effects of social mobility on levels of physical activity. One recent study of more than 2000 Australian adults age 26 to 36 years found that persistently high SEP and upward social mobility (indicated by educational level) from childhood to adulthood were associated with increased physical activity (13). Upward social mobility has also been found to be associated with decreased prevalence of physical inactivity in studies of health behaviors among Finnish adolescents (14) and older women in the UK (15). Better understanding the relationship between social mobility and physical activity may provide important insights into how social inequalities lead to poorer health.

The Medical Research Council National Survey of Health and Development is a nationally representative population-based birth cohort study that provides an opportunity to study the patterns of physical activity in a sample of more than 3800 men and women between age 31 and 53 years and relate them to SEP and inter-generational social mobility.

The aim of this work is to examine whether intergenerational change in SEP (as indicated by occupational class and educational attainment) was associated with differences in the types and patterns of physical activity, and if so, how.

## Methods

### Participants

The sample comprised National Survey of Health and Development participants, an initial sample of 2815 men and 2547 women followed since their birth in March 1946 (16). Medical and social data have been collected 23 times by home visits, medical examinations, and postal questionnaires.

### Measures

Self-reported information about physical activity was collected to differing extents at several sweeps of data collection. At ages 31, 36, and 43, a number of questions were asked about specific types of physical activity, and at age 53 a more general question was asked regarding sports, vigorous leisure activities, or exercises. In addition, at 36 years, more detailed information was collected, with study participants asked about the frequency and duration of participation in many different leisure time activities in the preceding month on the basis of the Minnesota leisure time physical activity questionnaire (17).

In the present analysis we focused on four different types of self-reported physical activity: (1) sedentary behavior during the working day; (2) walking during the working day and for pleasure; (3) cycling during the working day and for pleasure; and (4) LTPA. Sedentary behavior was examined at age 36 years; walking at 36 and 43 years; cycling at 31, 36, and 43 years; and LTPA at ages 36, 43 and 53. To summarize, categorical variables in each of these dimensions were derived at available ages on the basis of self-reported questionnaire information. A total of 16 response variables were used across the four types of physical activity, and 3847 study participants (71.7% of the original cohort) were included on at least one of these measures. By the beginning of the period considered in the present analysis, 6.0% of the original cohort had died, 9.7% had permanently refused, and 12.0% were living abroad (16).

Two different subdomains of SEP were examined: occupational class and educational attainment. Prospectively collected data were used to classify study members according to the occupational class of the head of the household at age 36 years and the occupational class of their father in 1950 (i.e., at age 4 years) on the basis of the British Registrar General's Social Classification (18): ‘I and II’, ‘III non-manual’, ‘IV manual’, or ‘IV and V’. Intergenerational occupational mobility was defined by combining ‘I and II’ with ‘III nonmanual’ (“nonmanual”) and ‘IV manual’ with ‘IV and V’ (“manual”) then defining the following four groups: ‘manual/manual’, ‘manual/nonmanual (upward)’, ‘nonmanual/manual (downward)’, and ‘nonmanual/nonmanual’.

Prospectively collected information on study members’ educational qualifications achieved by age 26 years were grouped into ‘no qualifications’, ‘lower secondary’ (‘O’-levels or equivalent, usually attained at 16 years), ‘advanced secondary’ (‘A’-levels or equivalent, usually attained at 18 years), and ‘degree-level or equivalent’. Father’s educational level, reported in 1952, was classified as ‘primary only’, ‘primary and further education (no qualifications attained)’, ‘secondary only or primary and further education or higher’, or ‘secondary and higher’. Intergenerational educational mobility was defined by combining the lower two classes in each education variable (‘lower’) and the more advance two classes (‘advanced’) then defining the following four groups: ‘lower/lower’, ‘lower/advanced (upward)’, ‘advanced/lower (downward)’, and ‘advanced/advanced’. Of the 3847 study members with at least one measure of physical activity, 77.7% had information on intergenerational occupational mobility and 84.5% had information on inter-generational educational mobility.

### Statistical Analyses

Different self-reported measures of physical activity were obtained at different time points, leading to complex, correlated data. Latent class analysis (LCA) was used to reduce the many derived measures of physical activity to a more useable form. LCA models identify a categorical latent (i.e., unobserved) class variable which is measured by a number of observed response variables. The objective is to identify the response variables that best distinguish between classes and to categorize people into their most likely classes given their observed responses (19).

A more detailed account of how the LCA was performed is available elsewhere (20). The purpose of the present paper is to use the previously derived latent classes, so only a brief description is given here. LCA was conducted separately for each type of physical activity (apart from sedentary behavior, for which data reduction was not required), and all participants with at least one measure of a given type of physical activity were included. Separate LCA models for males and females were used because these were found to give the best fit to the data. The most appropriate number of latent classes for each type of physical activity was determined with the use of several different measures of model fit (20). Posterior probabilities were derived by the LCA to quantify the probability with which an individual with given values for the response variables belonged to each latent class.

Logistic or multinomial logistic regression of one latent variable on another was used to examine pairwise associations between the latent variables for each type of physical activity. These analyses used robust standard errors and were weighted by LCA posterior probabilities to account for the uncertainty in class membership where appropriate. Associations between the latent variable for each type of physical activity and each measure of SEP were examined in the same manner. Analyses were repeated by use of the most likely latent class in unweighted logistic regressions for comparison.

Analyses were repeated by use of the study member's own occupational class at age 36 years (rather than the head of household's) and mother's educational level (rather than father's) as comparisons. Models were also fitted with adjustment for the season of data collection. Latent class analysis was performed with Mplus 6 (21), whereas (multinomial) logistic regression was conducted using Stata 11 (22).

## Results

For the majority of physical activity and SEP variables, there was strong (*p* < .001) evidence of a gender difference (Tables 1 and 2). The LCAs for walking, cycling, and LTPA included 3587, 3776, and 3671 study participants, respectively. The most appropriate number of latent classes was found to be two for walking (both males and females), two for cycling (both males and females), and three for LTPA (both males and females) (20).

More details regarding the interpretation of the latent classes are available elsewhere (20). To summarize, the two walking latent classes can be considered as ‘low’ (males 52.8% using estimated posterior class membership probabilities, females 33.5%) and ‘high’ (males 47.2%, females 66.5%) levels of activity; the two cycling classes as ‘low’ (males 91.4%, females 82.1%) and ‘high’ (males, 8.6%; females, 17.9%) levels of activity; and the three LTPA classes as ‘low’ activity (males 46.2%, females 48.2%), ‘gardening and do-it-yourself’ (males 22.8%, females 16.5%), and ‘sport and leisure’ (males 31.0%, females 35.3%).

In LCA the separation of the classes is often quantified in terms of entropy, which takes values between 0 and 1, with scores close to 1 indicating clearer classifications (23). The male walking classes (0.66) and cycling classes (0.87 and 0.64 for males and females, respectively) were clearly separated and the LTPA classes reasonably so (0.56 and 0.57), although entropy for female walking was low (0.37).

The three latent variables (walking, cycling, LTPA) and sedentary behavior at age 36 were associated with each other (Tables 3 and 4). Male respondents who reported being most sedentary during the working day at age 36 were much less likely to be in the high walking and cycling latent classes compared with those in the least sedentary group but more likely to be in the sport and leisure LTPA latent class. In females, only the association with walking latent class was observed. Males in the high walking latent class were less likely to be in the sport and leisure LTPA latent class compared with those in the low walking latent class. Both males and females in the high cycling latent class were more likely to be in the sport and leisure LTPA latent class.

Tables 5 and 6 show cross-tabulations of the physical activity latent variables with the SEP variables for males and females, respectively. For males, being a manual worker was relative to being a nonmanual worker, associated with lower levels of adult sedentary behavior during the working day (13.4% much sitting in classes IV and V compared with 43.6% in classes I and II), greater levels of walking (66.1% high compared with 32.8%), but also lower LTPA (24.3% sport and leisure compared with 39.4%). For female respondents, LTPA showed a similarly strong association, walking was somewhat less marked, and sedentary behavior showed a nonlinear association, with the III nonmanual class corresponding to the greatest level of adult sedentary behavior.

Similar patterns were observed for father’s occupational class in 1950, although differences between manual and nonmanual occupational classes were generally reduced. Compared with participants who remained in the manual occupational class, those from a similar background but who were upwardly mobile by age 36 reported more sedentary behavior during the working day and less walking in men only, and increased LTPA in both men and women. Compared with men who remained in the nonmanual occupational class, men who were downwardly mobile reported less sedentary behavior, more walking, and less LTPA. In women whose fathers were nonmanual occupational class there were similar patterns, although the magnitudes of the differences were reduced.

In women who were nonmanual occupational class at age 36, there were residual differences in LTPA between those with manual and nonmanual occupational class fathers. This effect also was observed in those who were manual occupational class at age 36.

Although the effects of occupational class on physical activity were most often seen as a manual/nonmanual split, the effects of educational class were more linear. In both men and women, having more advanced educational qualifications was associated with increased sedentary behavior during the working day and decreased walking, but also with increased LTPA. Similar patterns were observed for study members' father's educational level, although the magnitudes of the associations were generally reduced.

Those with upward intergenerational mobility into the advanced educational class reported more sedentary behavior during the day and less walking but more LTPA. Similarly, study participants demonstrating downward educational mobility reported less sedentary behavior during the working day and more walking (men only) but less LTPA (women only).

There was some evidence of a residual effect of father's educational class. Among study members of advanced educational class, having a father of advanced rather than lower educational class led to increased sedentary behavior during the working day (men only), reduced walking (men only), and increased LTPA (women only). A similar residual effect was seen for sedentary behavior in male study members of lower educational class.

Repeating the analysis using most likely latent classes in unweighted logistic regressions made little difference to the percentage of study participants corresponding to each level of SEP and did not affect the conclusions drawn (results not shown).

In models with adjustment for the effects of seasonal variation of physical activity the estimated associations changed very little (results not shown).

Repeating the analyses using women's own occupational class at age 36 led to an amplification of the effects of occupational class and intergenerational occupational mobility on sedentary behavior and, to a lesser extent, walking in women, although the LTPA results were essentially unchanged (results not shown). When mother's rather than father's educational level was used (and intergenerational educational mobility defined on this basis), the direction and overall strength of associations were generally very similar (results not shown).

## Discussion

In a large, population-based, prospective study we found SEP and intergenerational social mobility to be associated with previously identified latent class variables for different types of physical activity and an additional observed variable for sedentary behavior. Manual occupational classes and lower educational classes, both for the study member and their father, were associated with lower levels of sedentary behavior during the working day and greater levels of walking activity, most likely through the subject having a type of job that requires more walking. Greater levels of LTPA (particularly sport and leisure activity) were found to be more common in those of nonmanual occupational class and those with more advanced educational qualifications, most likely as a conscious compensation for the detrimental effect on their health of having a more sedentary occupation.

The large differences in physical activity generally found between study members whose SEP (occupational or educational class) changed from their father's and study members whose SEP remained the same as their father's suggests that it was largely their own SEP that determined their pattern of physical activity rather than their parents’, illustrating the positive potential of social mobility. However, the residual effect of father's SEP in those with the same SEP in adulthood suggests that when SEP changes between generations, it may take further generations before the full implications are felt.

Our findings suggest that it is important to consider several types of activity rather than extrapolating from only one in studies of physical activity. We cannot be certain whether doing more LTPA (generally those of greater SEP) amounts to more total physical activity than being less sedentary and walking more (lower SEP). People who are particularly active during their working day may well be too tired to engage in greater levels of activity in their leisure time.

The observed associations were often less clear in female respondents. Although this may be attributable to less distinct separation of the latent classes (21), it may also indicate that using the occupational class of the head of household (usually a male) at age 36 years is a relatively poorer measure of SEP in women, leading to attenuation. Using women's own occupational class at age 36 led to stronger associations with sedentary behavior and, to a lesser extent, walking. Although women's own occupational class may naturally be more strongly associated with occupation-based physical activity—sedentary behavior was based on time sitting down during the day—head of household's occupational class is likely to provide a more reliable general measure of SEP at age 36 years because many women in this cohort were at home looking after children.

The data used in the present analysis were collected from 1977 to 1999 and secular trends in physical activity and women’s employment may mean that the relationships observed in this cohort have changed in later cohorts. In recent years, decreases in occupational physical activity coupled with an upward trend in sports participation have been noted in the UK (24). In addition, the increase in the female labor market (25) is likely to have led to more similar patterns of occupational activity across the sexes.

The acknowledged association between greater SEP and greater levels of leisure-time or moderate-vigorous activity (11) was clearly replicated in our study. Cleland et al. (13) found that persistently high SEP and upward social mobility from childhood to adulthood were associated with increases in physical activity. Although our study did not allow us to examine changes in physical activity, we found that high SEP in childhood or adult life, or upward intergenerational social mobility were associated with greater levels of LTPA. However, we also found these groups to correspond to lower levels of walking and greater sedentary behavior.

In addition, Cleland et al. found that childhood SEP had no lasting impact on physical activity levels once adult SEP was taken into account. Similar findings have been reported in other studies (26, 27). In our analysis, however, we found a residual effect of father’s SEP for some types of physical activity. In a Dutch prospective cohort of 25- to 74–year-old subjects, van de Mheen et al. (28) similarly found childhood SEP to be associated with frequent physical activity after adjustment for current SEP, although only in female subjects.

There is much strength to this analysis. Several different physical activities were examined and the concordance of our conclusions using two different subdomains of SEP suggests our findings are robust. The LCA approach identified clearly separated latent classes which provided a good fit to the data, although for walking in females the separation was less clear.

All study participants with at least one nonmissing variable within a given type of physical activity were included in that LCA under the assumption of missing at random. The missing at random assumption is difficult to assess but seems reasonable given the strong correlations observed between most variables within the same type of physical activity (20).

A second missing data assumption is that study participants excluded from the final logistic regression models can be considered missing completely at random. As with any long-running cohort study, some attrition as the result of deaths and emigration is unavoidable, and avoidable loss as the result of refusal and failure to trace is relatively low in this study (29). The effective sample sizes ranged between 2821 and 3564, or between 72.8% and 92.0% of those alive and still living in the UK at the start of follow-up for this study. Exclusion was found to be associated with educational level for several of the physical activity-SEP combinations, with lower educational level resulting in increased exclusion, reflecting the greater attrition at lower educational levels previously reported in this cohort (29). However, no associations were found between exclusion and other SEP variables. The effective sample sizes compare favorably with the number of study participants successfully contacted at each data collection (16). Because the 3035 study participants successfully contacted at age 53 have been found to be broadly representative of native-born adults living in England, Scotland, and Wales at the time of data collection (29), we are confident that our samples were similarly broadly representative.

However, there are also limitations. Data availability determined at what ages and to what extent we could examine different types of physical activity, with only a single measure of sedentary behavior being available. Measures of physical activity obtained from questionnaires may be prone to nondifferential measurement error (30). The retrospectively self-reported measures may have led to recall bias, potentially differentially through social desirability and approval influencing the responses (31). Although the physical activity data were almost always collected between spring and autumn, misclassification caused by seasonal variability of activity behaviors (32) may have been present. However, adjustment for the season of data collection made very little difference to the estimated associations.

In addition, some of the physical activity items may be differentially relevant to people in different socioeconomic groups. For example, those of lower SEP may be less likely to have homes with gardens, so would by necessity do less gardening. This may partially confound apparent social differences in physical activity (33).

This descriptive analysis has made no attempt to disentangle the complex relationships between socioeconomic position, physical activity, and the many potential confounding or mediating variables between the two, such as health status, mobility limitation, and obesity. Each of these could be considered as either a cause or an effect of low levels of physical activity, and a rigorous investigation of these issues is beyond the scope of the present analysis. As such, we cannot rule out the possibility that the observed associations may be at least partly the result of unmeasured confounders.

An alternative approach to that used in the present analysis would have been to include all the physical activity response variables in a single LCA to derive overarching physical activity latent classes. We decided against this approach because we wanted to capture specific types of physical activity that would also be applicable to different settings and to maintain comparability with other cohorts, as most studies concentrate on a single type of physical activity.

In conclusion, this study found clear evidence of social differences in different types of physical activity. Persistently high SEP and upward social mobility were associated with greater levels of LTPA but also with greater levels of sedentary behavior during the working day and less walking. In addition, the lack of strong correlation between most of the types of physical activity suggests that studies examining relationships between physical activity and health should consider many types of activity rather than extrapolating from only one.

## Figures and Tables

**Table 1:** Physical activity variables in the Medical Research Council National Survey of Health and Development

Physical activity variable	Males		Females		Total	
	n	%	n	%	n	%
Sedentary behavior						
Time sitting down during day at age 36 years∗						
More than half to practically all the time	534	32.6	333	20.3	867	26.4
Less than to about half the time	586	35.7	545	33.2	1131	34.5
Almost none of the time	520	31.7	765	46.6	1285	39.1
Total	1640		1643		3283	
Walking						
Age 36 years						
Time spent walking during day∗						
Less than half the time	747	46.1	497	30.5	1244	38.3
At least half the time	512	31.6	594	36.5	1106	34.0
Practically all the time	363	22.4	538	33.0	901	27.7
Total	1622		1629		3251	
Time spent walking to work∗						
<5 minutes	1219	80.2	679	67.4	1898	75.1
5–15 minutes	224	14.7	249	24.7	473	18.7
16+ minutes	77	5.1	79	7.9	156	6.2
Total	1520		1007		2527	
Time spent walking for pleasure in last month†						
0 hours	583	35.9	492	30.0	1075	33.0
1–6 hours	538	33.2	583	35.6	1121	34.4
> 6 hours	502	30.9	564	34.4	1066	32.7
Total	1623		1639		3262	
Age 43 years						
Distance walked on average weekday∗						
≤0.5 miles	493	30.8	611	38.2	1104	34.5
0.5–2.5 miles	709	44.2	785	49.0	1494	46.6
>2.5 miles	401	25.0	205	12.8	606	18.9
Total	1603		1601		3204	
Cycling						
Age 31 years						
Frequency of cycling						
Seldom or never	780	79.0	830	82.2	1610	80.6
Less than once a week	106	10.7	105	10.4	211	10.6
At least once a week	102	10.3	75	7.4	177	8.9
Total	988		1010		1998	
Age 36 years						
Time spent cycling per week†						
0 minutes	1334	80.8	1392	83.6	2726	82.2
1–99 minutes to work or 1–59 minutes outside work	134	8.1	102	6.1	236	7.1
100+ minutes to work or 60+ minutes outside work	184	11.1	171	10.3	355	10.7
Total	1652		1665		3317	
Age 43 years						
Distance cycled on average weekday∗						
0 miles	1390	87.1	1384	86.6	2774	86.9
0.1–1.5 miles	67	4.2	120	7.5	187	5.9
>1.5 miles	139	8.7	94	5.9	233	7.3
Total	1596		1598		3194	
Leisure time physical activity						
Age 36 years						
Gardening∗						
Inactive	334	20.9	432	26.5	766	23.7
Less active	538	33.7	684	42.0	1222	37.9
Most active	724	45.4	514	31.5	1238	38.4
Total	1596		1630		3226	
DIY∗						
Inactive	506	31.7	987	59.9	1493	46.0
Less active	458	28.7	402	24.4	860	26.5
Most active	631	39.6	259	15.7	890	27.4
Total	1595		1648		3243	
Sport or leisure activities∗						
Inactive	512	32.2	706	43.8	1218	38.0
Less active	466	29.3	525	32.5	991	30.9
Most active	614	38.6	382	23.7	996	31.1
Total	1592		1613		3205	
Age 43 years						
Vigorous housework or cleaning∗						
Inactive	1146	71.3	429	26.7	1575	49.0
Less active	356	22.2	537	33.4	893	27.8
Most active	105	6.5	642	39.9	747	23.2
Total	1607		1608		3215	
Heavy gardening∗						
Inactive	860	54.3	1101	68.9	1961	61.6
Less active	412	26.0	302	18.9	714	22.4
Most active	311	19.6	196	12.3	507	15.9
Total	1583		1599		3182	
Heavy building or DIY∗						
Inactive	1153	75.0	1522	95.4	2675	85.4
Less active	203	13.2	47	2.9	250	8.0
Most active	182	11.8	26	1.6	208	6.6
Total	1538		1595		3133	
Sports or vigorous leisure activities∗						
Inactive	774	48.6	888	55.4	1662	52.0
Less active	315	19.8	455	28.4	770	24.1
Most active	503	31.6	259	16.2	762	23.9
Total	1592		1602		3194	
Age 53 years						
Regular vigorous physical activity						
Inactive	705	48.1	772	50.9	1477	49.5
Less active	434	29.6	397	26.2	831	27.9
Most active	326	22.3	349	23.0	675	22.6
Total	1465		1518		2983	

DIY = do-it-yourself. All % are column percentages.

∗ χ^2^ test for difference between males and females: *p* < .001.

† χ^2^ test for difference between males and females: 0.001 ≤ *p* < .05.

**Table 2:** Socioeconomic position variables in subjects who have data for at least one dimension of physical activity in the Medical Research Council National Survey of Health and Development

Socioeconomic position variable	Males (n = 1940)		Females (n = 1907)		Total (n = 3847)	
	n	%	n	%	n	%
Head of household’s occupational class at age 36∗						
I and II	729	44.9	623	39.6	1352	42.3
III nonmanual	166	10.2	257	16.3	423	13.2
III manual	523	32.2	429	27.3	952	29.8
IV and V	204	12.6	265	16.8	469	14.7
Total	1622		1574		3196	
Father’s occupational class in 1950						
I and II	405	22.8	392	22.7	797	22.7
III nonmanual	329	18.5	328	19.0	657	18.7
III manual	540	30.4	531	30.7	1071	30.5
IV and V	504	28.3	478	27.6	982	28.0
Total	1778		1729		3507	
Intergenerational occupational mobility						
Manual/manual	519	34.7	474	32.6	993	33.7
Manual/nonmanual (upward)	359	24.0	368	25.3	727	24.7
Nonmanual/manual (downward)	144	9.6	174	12.0	318	10.8
Nonmanual/nonmanual	475	31.7	436	30.0	911	30.9
Total	1497		1452		2949	
Educational qualifications achieved by age 26∗						
No qualifications	712	39.0	694	38.7	1406	38.9
Lower secondary	370	20.3	616	34.3	986	27.3
Advanced secondary	486	26.6	395	22.0	881	24.4
Degree level	256	14.0	89	5.0	345	9.5
Total	1824		1794		3618	
Father’s education						
Primary only	959	56.4	979	58.2	1938	57.3
Primary and further education (no qualifications attained)	242	14.2	212	12.6	454	13.4
Secondary only or primary and further education or higher	235	13.8	225	13.4	460	13.6
Secondary and greater	265	15.6	267	15.9	532	15.7
Total	1701		1683		3384	
Inter-generational educational mobility∗						
Lower/lower	804	49.4	979	60.4	1783	54.9
Lower/advanced (upward)	343	21.1	170	10.5	513	15.8
Advanced/lower (downward)	169	10.4	210	12.9	379	11.7
Advanced/advanced	312	19.2	262	16.2	574	17.7
Total	1628		1621		3249	

All % are column percentages.

∗ χ^2^ test for difference between males and females: *p* < .001.

**Table 3:** Associations between physical activity (latent) variables in the Medical Research Council National Survey of Health and Development (males)

	Walking latent class				Cycling latent class				Leisure time physical activity latent class				
	n	Low (%)	High (%)	LRT *p*	n	Low (%)	High (%)	LRT *p*	n	Low (%)	Gardening and DIY (%)	Sport and leisure (%)	LRT *p*
Sedentary behavior at age 36 years													
Much sitting	1640	98.3	1.7	<.001	1639	94.3	5.7	.003	1638	38.7	22.1	39.2	<.001
Average sitting		51.5	48.5			91.1	8.9			43.1	23.7	33.2	
Little sitting		18.5	81.5			89.5	10.5			50.1	23.8	26.1	
Walking latent class													
Low					1794	92.3	7.7	.04	1795	40.4	23.0	36.7	.001
High						90.2	9.8			49.5	23.0	27.5	
Cycling latent class													
Low									1807	45.3	22.6	32.1	<.001
High										33.8	27.5	38.7	

DIY = do-it-yourself; LRT = likelihood ratio test. All % are row percentages.

**Table 4:** Associations between physical activity (latent) variables in the Medical Research Council National Survey of Health and Development (females)

	Walking latent class				Cycling latent class				Leisure time physical activity latent class				
	n	Low (%)	High (%)	LRT *p*	n	Low (%)	High (%)	LRT *p*	n	Low (%)	Gardening and DIY (%)	Sport and leisure (%)	LRT *p*
Sedentary behavior at age 36 years													
Much sitting	1643	59.3	40.7	<.001	1643	81.1	18.9	.38	1643	48.6	13.8	37.5	.02
Average sitting		42.3	57.7			82.1	17.9			47.3	14.5	38.2	
Little sitting		18.7	81.3			79.6	20.4			44.5	19.3	36.2	
Walking latent class													
Low					1788	81.8	18.2	.23	1790	46.6	14.7	38.8	.03
High						80.3	19.7			45.6	17.7	36.7	
Cycling latent class													
Low									1793	48.3	15.9	35.8	<.001
High										36.2	19.6	44.2	

DIY = do-it-yourself; LRT = likelihood ratio test. All % are row percentages.

**Table 5:** Associations between physical activity (latent) variables and socioeconomic position in the Medical Research Council National Survey of Health and Development (males)

	Sedentary behavior (age 36 years)				Walking (ages 36 and 43 years)			Cycling (ages 31, 36, and 43 years)			LTPA (age 36, 43, and 53 years)			
	n	Much sitting %	Average sitting %	Little sitting %	n	Low %	High %	n	Low %	High %	n	Low %	Gardening and DIY %	Sport and leisure %
Head of household’s occupational class at age 36 years														
I and II	727	43.6	39.9	16.5	729	67.2	32.8	729	92.0	8.0	727	35.9	24.7	39.4
III nonmanual	164	50.6	39.6	9.8	166	72.5	27.5	166	93.2	6.8	166	38.7	23.4	37.9
III manual	521	19.2	28.8	52.0	523	45.0	55.0	523	91.6	8.4	523	49.4	24.3	26.3
IV and V	201	13.4	34.8	51.7	204	33.9	66.1	204	88.1	11.9	204	58.8	16.9	24.3
N	1613				1622			1622			1620			
LRT *p*	<.001				<.001			.17			<.001			
LRT *p* (trend)	<.001				<.001			.14			<.001			
Father’s occupational class in 1950														
I and II	346	41.3	35.3	23.4	375	65.1	34.9	399	90.3	9.7	384	39.9	22.4	37.7
III nonmanual	277	45.1	35.7	19.1	309	67.0	33.0	322	94.1	5.9	317	37.7	25.0	37.3
III manual	465	26.9	35.9	37.2	510	52.4	47.6	534	92.8	7.2	516	45.1	23.7	31.2
IV and V	427	24.6	36.3	39.1	453	47.5	52.5	489	89.3	10.7	476	51.9	22.6	25.5
N	1515				1647			1744			1693			
LRT *p*	<.001				<.001			.01			<.001			
LRT *p* (trend)	<.001				<.001			.41			<.001			
Intergenerational occupational social mobility														
Manual/Manual	516	17.1	29.7	53.3	519	40.7	59.3	519	90.9	9.1	519	53.4	21.8	24.9
Manual/nonmanual (upward)	359	38.4	45.1	16.4	359	63.5	36.5	359	91.7	8.3	358	38.0	26.8	35.2
Nonmanual/manual (downward)	143	20.3	35.0	44.8	144	49.4	50.6	144	90.0	10.0	144	46.3	25.9	27.7
Nonmanual/nonmanual	471	50.3	35.5	14.2	475	72.6	27.4	475	93.1	6.9	474	35.1	23.4	41.5
N	1489				1497			1497			1495			
LRT *p*	<.001				<.001			.40			<.001			
Educational qualifications achieved by age 26 years														
No qualifications	585	18.5	33.3	48.2	646	40.6	59.4	698	91.0	9.0	668	54.8	20.7	24.5
Lower secondary	332	31.6	38.9	29.5	352	58.5	41.5	363	91.0	9.0	360	44.1	21.8	34.1
Advanced secondary	427	38.2	37.7	24.1	463	62.9	37.1	480	92.7	7.3	469	37.3	26.5	36.2
Degree level	219	58.9	35.2	5.9	236	80.0	20.0	252	88.6	11.4	241	29.4	27.1	43.5
N	1563				1697			1793			1738			
LRT *p*	<.001				<.001			.18			<.001			
LRT *p* (trend)	<.001				<.001			.68			<.001			
Father’s educational level														
*Primary only*	813	25.0	36.2	38.9	884	49.4	50.6	937	91.8	8.2	913	48.7	23.0	28.3
Primary and further education (no qualifications attained)	219	34.2	35.6	30.1	229	59.1	40.9	238	90.9	9.1	233	40.9	24.9	34.2
Secondary only or primary and further education or greater	204	39.2	40.2	20.6	221	62.9	37.1	233	90.7	9.3	225	41.1	24.9	34.0
Secondary and greater	219	48.9	38.4	12.8	244	71.0	29.0	260	91.9	8.1	250	35.4	21.9	42.8
N	1455				1578			1668			1621			
LRT *p*	<.001				<.001			.90			<.001			
LRT *p* (trend)	<.001				<.001			.81			<.001			
Intergenerational educational social mobility														
Lower/lower	688	21.8	34.3	43.9	739	45.7	54.3	788	91.1	8.9	763	51.9	21.5	26.6
Lower/advanced (upward)	306	37.3	40.2	22.5	328	63.5	36.5	338	92.4	7.6	332	36.3	28.6	35.1
Advanced/lower (downward)	145	29.7	44.1	26.2	159	54.4	45.6	166	92.9	7.1	163	45.3	20.6	34.1
Advanced/advanced	264	52.3	36.7	11.0	287	74.6	25.4	308	90.4	9.6	293	33.1	25.1	41.8
N	1403				1513			1600			1551			
LRT *p*	<.001				<.001			.61			<.001			

LRT = likelihood ratio test; LTPA = leisure-time physical activity. All % are row percentages.

**Table 6:** Associations between physical activity (latent) variables and socioeconomic position in the Medical Research Council National Survey of Health and Development (females)

	Sedentary behaviour (age 36 years)				Walking (ages 36 and 43 years)			Cycling (ages 31, 36, and 43 years)			LTPA (age 36, 43, and 53 years)			
	n	Much sitting (%)	Average sitting (%)	Little sitting (%)	n	Low (%)	High (%)	n	Low (%)	High (%)	n	Low (%)	Gardening and DIY (%)	Sport and leisure (%)
Head of household’s occupational class at age 36 years														
I and II	613	18.1	36.4	45.5	623	38.4	61.6	623	80.6	19.4	623	36.6	17.0	46.4
III nonmanual	256	36.7	25.8	37.5	257	37.0	63.0	257	81.7	18.3	257	49.3	13.6	37.1
III manual	425	21.6	31.5	46.8	429	33.2	66.8	429	79.1	20.9	429	52.0	17.2	30.8
IV and V	262	12.2	28.6	59.2	265	25.3	74.7	265	83.6	16.4	265	54.5	17.9	27.6
N	1556				1574			1574			1574			
LRT *p* value	<.001				<.001			.35			<.001			
LRT *p* value (trend)	.005				<.001			.58			<.001			
Father’s occupational class in 1950														
I and II	341	19.6	36.7	43.7	373	37.2	62.8	387	77.7	22.3	389	33.8	18.3	47.9
III nonmanual	287	25.1	33.1	41.8	308	40.0	60.0	326	79.7	20.3	311	36.7	19.6	43.7
III manual	467	20.3	29.8	49.9	500	33.2	66.8	524	83.7	16.3	508	50.7	16.3	33.0
IV and V	415	17.6	33.7	48.7	451	30.6	69.4	469	80.6	19.4	459	55.8	14.2	30.0
N	1510				1632			1706			1657			
LRT *p*	.08				.004			.05			<.001			
LRT *p* (trend)	.14				.002			.09			<.001			
Intergenerational occupational social mobility														
Manual/manual	469	17.7	30.7	51.6	474	29.0	71.0	474	81.9	18.1	474	57.5	16.0	26.5
Manual/nonmanual (upward)	364	22.3	30.5	47.3	368	34.8	65.2	368	82.5	17.5	368	48.3	13.8	37.9
Nonmanual/manual (downward)	172	18.6	31.4	50.0	174	33.8	66.2	174	77.6	22.4	174	40.0	20.7	39.3
Nonmanual/nonmanual	430	24.9	35.6	39.5	436	40.8	59.2	436	78.8	21.2	436	32.9	18.7	48.4
N	1435				1452			1452			1452			
LRT *p*	.01				<.001			.19			<.001			
Educational qualifications achieved by age 26 years														
No qualifications	600	14.7	31.5	53.8	655	28.4	71.6	683	81.8	18.2	665	57.1	15.9	27.0
Lower secondary	543	28.0	27.1	44.9	578	36.0	64.0	611	81.4	18.6	586	45.2	17.5	37.3
Advanced secondary	349	14.9	44.4	40.7	347	40.3	59.7	391	79.9	20.1	379	30.7	17.2	52.1
Degree level	77	26.0	42.9	31.2	81	50.3	49.7	86	72.7	27.3	84	28.6	13.5	57.9
N	1569				1688			1771			1714			
LRT *p*	<.001				<.001			.11			<.001			
LRT *p* (trend)	<.001				<.001			.05			<.001			
Father’s educational level														
Primary only	855	20.1	30.8	49.1	919	32.0	68.0	963	82.3	17.7	935	52.9	15.7	31.4
Primary and further education (no qualifications attained)	183	23.5	32.8	43.7	195	35.8	64.2	211	78.9	21.1	199	43.5	18.4	38.1
Secondary only or primary and further education or higher	201	19.9	34.3	45.8	217	37.6	62.4	225	79.3	20.7	217	36.8	19.4	43.8
Secondary and higher	226	20.4	38.9	40.7	251	40.8	59.2	262	77.8	22.2	255	31.1	16.6	52.3
N	1465				1582			1661			1606			
LRT *p*	.25				.006			.14			<.001			
LRT *p* (trend)	.04				<.001			.03			<.001			
Intergenerational educational social mobility														
Lower/lower	854	20.4	28.6	51.1	917	30.6	69.4	965	82.0	18.0	933	53.4	16.2	30.3
Lower/advanced (upward)	152	18.4	48.0	33.6	158	42.7	57.3	167	79.5	20.5	162	37.9	15.9	46.2
Advanced/lower (downward)	181	27.1	30.4	42.5	200	36.3	63.7	208	81.0	19.0	201	44.0	18.9	37.2
Advanced/advanced	231	15.2	42.0	42.9	249	42.0	58.0	259	75.8	24.2	252	24.5	17.2	58.3
N	1418				1524			1599			1548			
LRT *p*	<.001				<.001			.07			<.001			

LRT = likelihood ratio test; LTPA = leisure-time physical activity. All % are row percentages.
